# Supplementary material for: Repeatability of protein structural evolution following convergent gene fusions
Source: Nat Commun. 2025 Sep 22;16:8278. doi: 10.1038/s41467-025-63898-x (PMC12454647; doi:10.1038/s41467-025-63898-x)
Supplement: Supplementary file 1 — Supplementary Information [file 41467_2025_63898_MOESM1_ESM.pdf]

Supplementary Information

Repeatability of protein structural evolution following convergent gene fusions

Naoki Konno*[1], Keita Miyake[2], Satoshi Nishino[3,4], Kimiho Omae[3,5], Haruaki Yanagisawa[6], Saburo Tsuru[7], Yuki Nishimura[3], Masahide Kikkawa[6], Chikara Furusawa[7, 8], Wataru Iwasaki[1,3]

1) Department of Biological Sciences, Graduate School of Science, The University of Tokyo, 7-3-1 Hongo, Bunkyo-ku, Tokyo 113-0033, Japan.
2) Department of General Systems Studies, Graduate School of Arts and Sciences, The University of Tokyo, 3-8-1 Komaba, Meguro-ku, Tokyo 153-8902, Japan.
3) Department of Integrated Biosciences, Graduate School of Frontier Sciences, The University of Tokyo, 5-1-5 Kashiwanoha, Kashiwa, Chiba 277-0882, Japan.
4) Atmosphere and Ocean Research Institute, The University of Tokyo, 5-1-5 Kashiwanoha, Kashiwa, Chiba, 277-0882, Japan.
5) RIKEN Cluster for Pioneering Research, RIKEN, 2-1 Hirosawa, Wako, Saitama, 351-0198, Japan
6) Department of Cell Biology and Anatomy, Graduate School of Medicine, The University of Tokyo, 7-3-1 Hongo, Bunkyo-ku, Tokyo 113-0033, Japan.
7) Universal Biology Institute, The University of Tokyo, 7-3-1 Hongo, Bunkyo-ku, Tokyo 113-0033, Japan.
8) RIKEN Center for Biosystems Dynamics Research, RIKEN, 6-2-3 Furuedai, Suita, Osaka, 565-0874, Japan.

*Correspondence should be addressed to N. K. (konno-naoki555@g.ecc.u-tokyo.ac.jp)

This PDF file includes:

1. **Supplementary Figures 1-12**
2. **Original gel images**

We also provide the following supplementary materials as separated files:

3. **Supplementary Video 1**
   Movies of molecular dynamics for AdhE dimer, AdhE hexamer, BdhE dimer, and BdhE tetramer. Movies for Run 1 in the three replication experiments are provided.
4. **Supplementary Data 1-3**
   Supplementary Data 1. Multiple sequence alignment of BdhE, AdhE, and sister-clade single-domain families of them.
   Supplementary Data 2, 3. Multiple sequence alignments used for gene phylogeny reconstructions of ALDH- (Data 2) and ADH- (Data 3) containing proteins.

Supplementary Figures

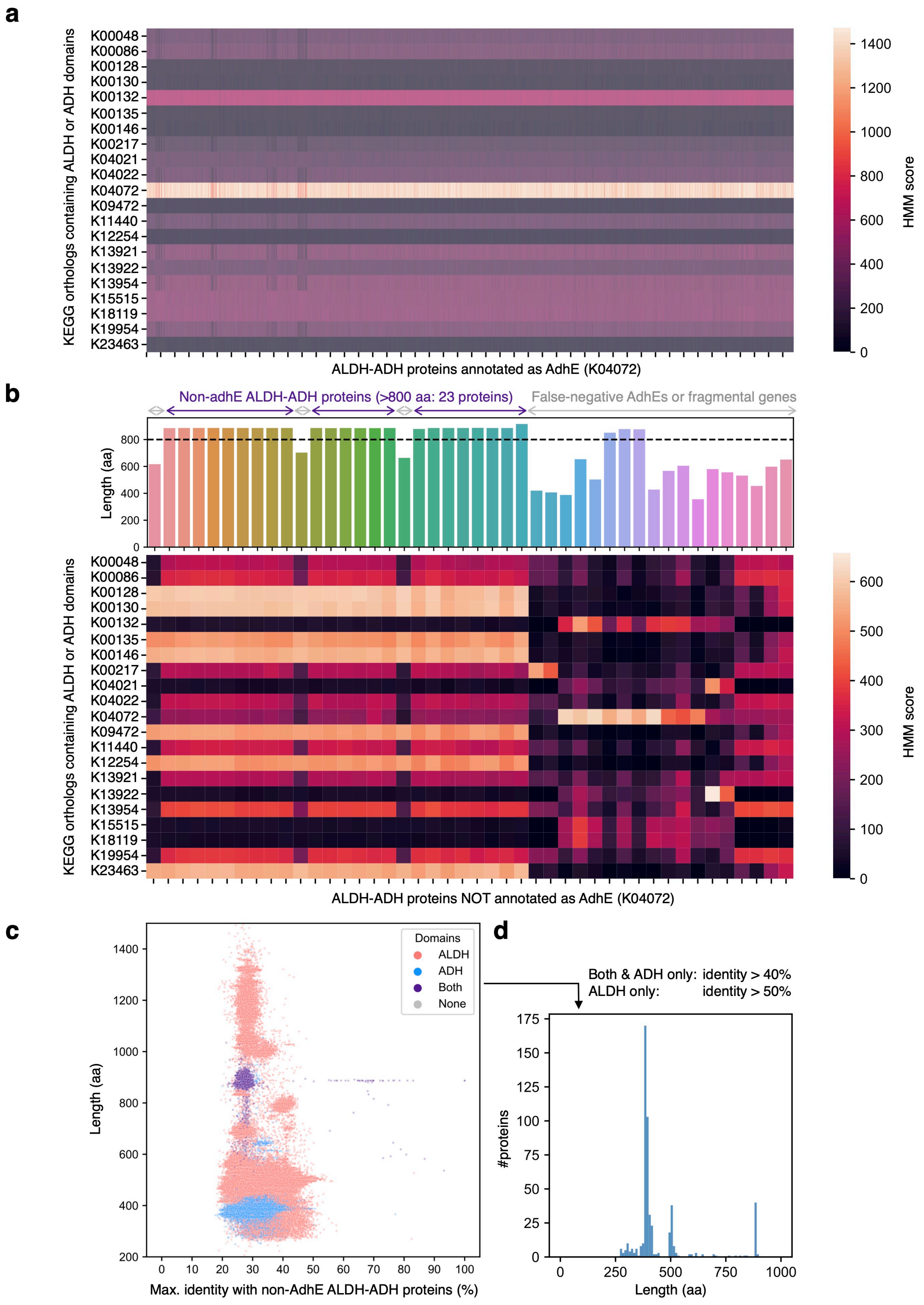

Supplementary Figure 1. *In silico* extraction of non-AdhE ALDH-ADH fusion proteins. a The results of ortholog annotation for UniProt protein entries annotated as AdhE (K04072) by KofamScan. The heatmap shows Hidden Markov Model (HMM) scores for every AdhE protein and every KEGG Ortholog possessing ALDH or ADH domains. As expected, K04072 showed the highest HMM scores overall. **b** The results of ortholog annotation by KofamScan for bacterial proteins NOT annotated as AdhE. The heatmap represents the HMM scores in the same way as **Supplementary Fig. 1a**. The bar plot indicates the amino acid length of each protein. As shown in the panel, we focused on the 23 proteins showing >800 aa length and highest HMM scores for non-AdhE orthologs as full-length non-AdhE fusion proteins with ALDH and ADH domains. **c** The profiles of the search hit proteins of sequence-similarity-based search by querying the 23 proteins in (b) against all the proteins coded in 45,555 bacterial genomes. Each dot represents each search hit protein, and the X and Y axis represents the maximum alignment identity with query sequences and the amino acid length, respectively. **d** The histogram of amino acid length after extracting search hit proteins based on maximum alignment identity. The extraction threshold of alignment identities was 40% and 50% for proteins with both domains or only ADH domain and for those with only ALDH domain, respectively.

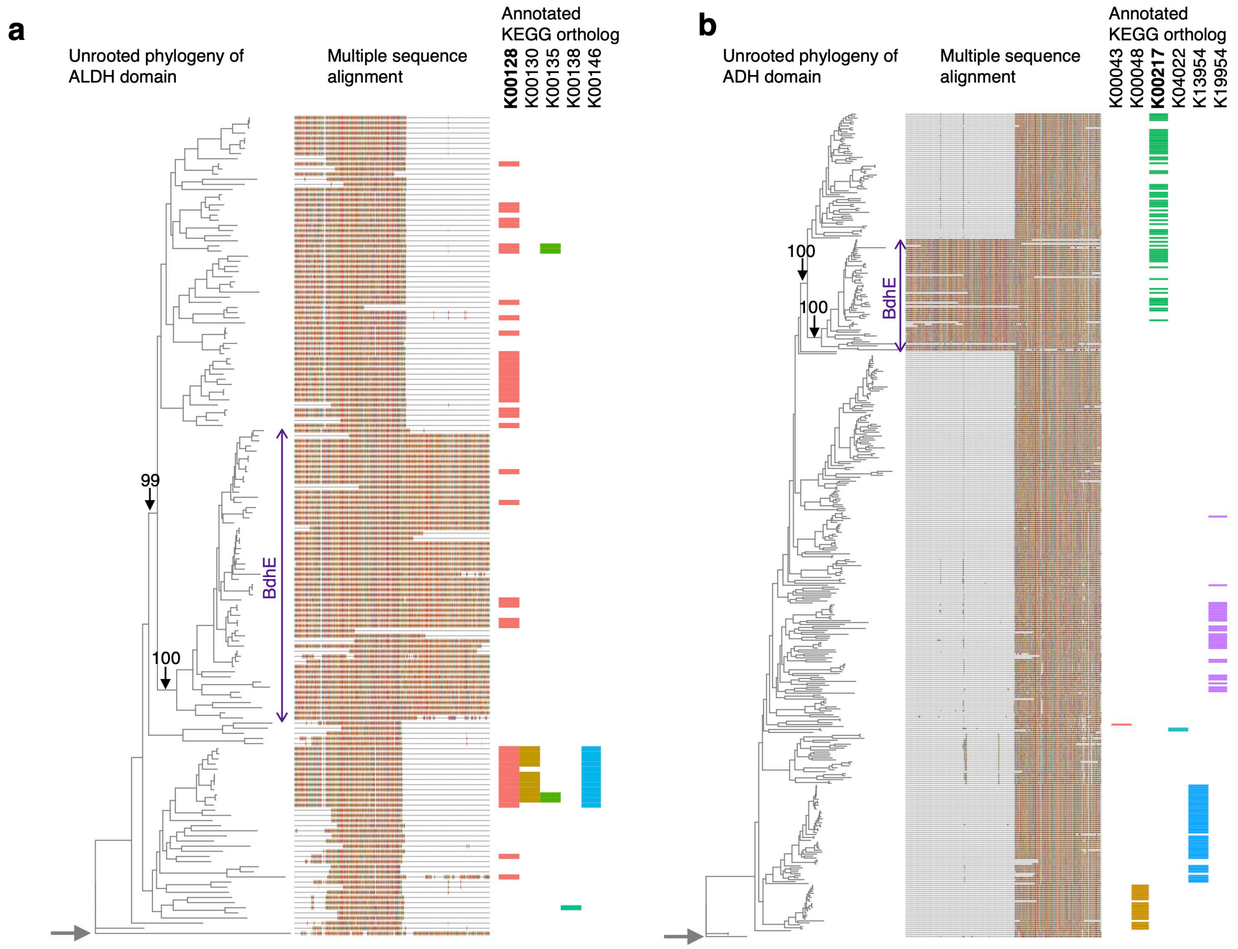

Supplementary Figure 2. Domain-wise gene phylogenies around non-AdhE ALDH-ADH fusion proteins. Unrooted phylogenetic trees and multiple sequence alignments (MSAs) of the sequence-similarity search hit proteins (**Supplementary Fig. 1d**) with ALDH (a) or ADH (b) domain as well as an AdhE of *Escherichia coli*. *E. coli*'s AdhE is represented by the grey arrow. Ultrafast bootstrap values are shown for specific branches with black arrows. Annotated KEGG Orthologs are indicated along with the phylogenies and MSAs.

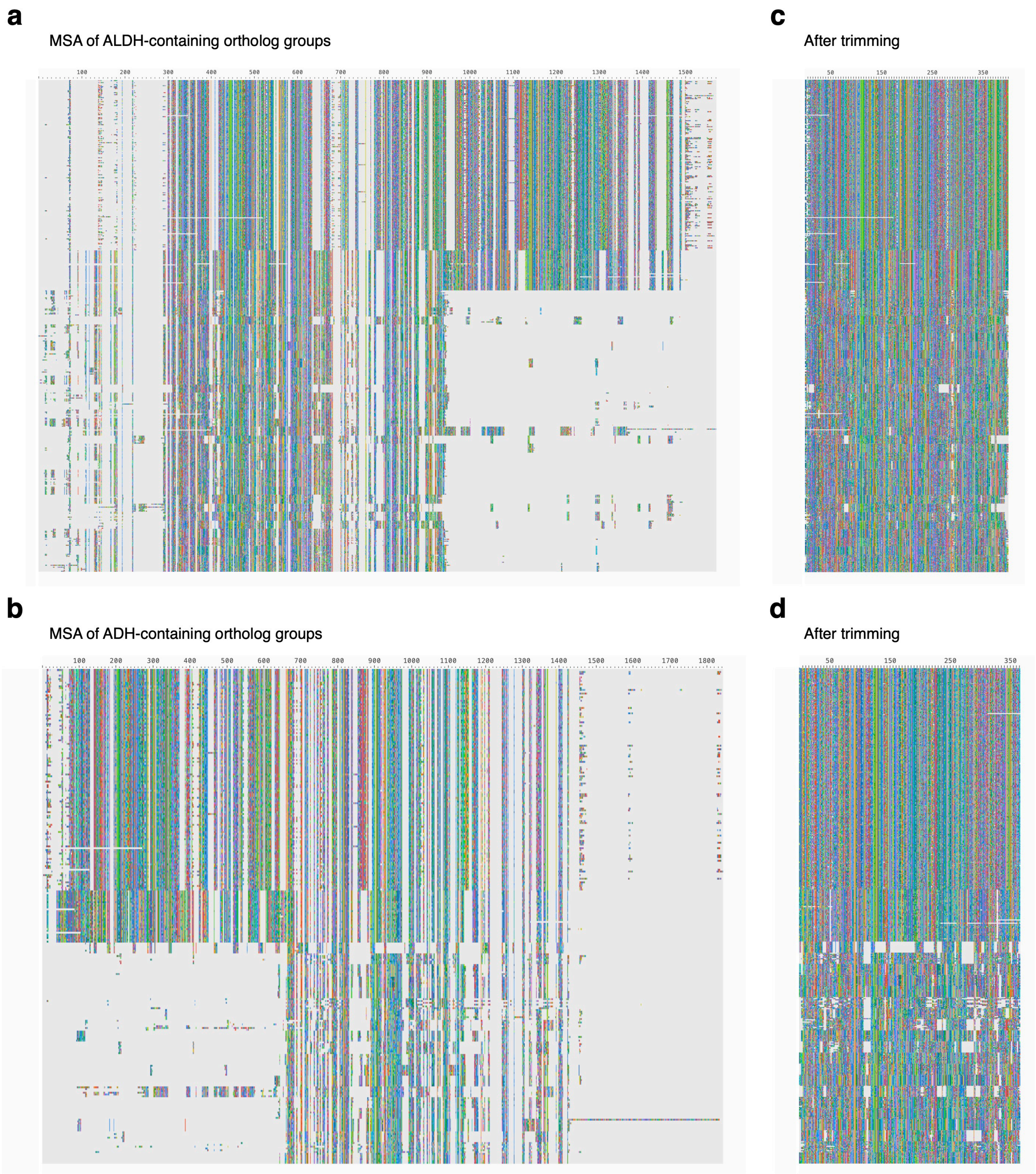

Supplementary Figure 3. Multiple sequence alignments for the phylogenetic analyses across ALDH and ADH families. a, b Multiple sequence alignments of ALDH- (a) and ADH- (b) containing proteins by MAFFT (*1*). **c, d** Multiple sequence alignments of ALDH- (c) and ADH- (d) after trimming by TrimAl (*2*).

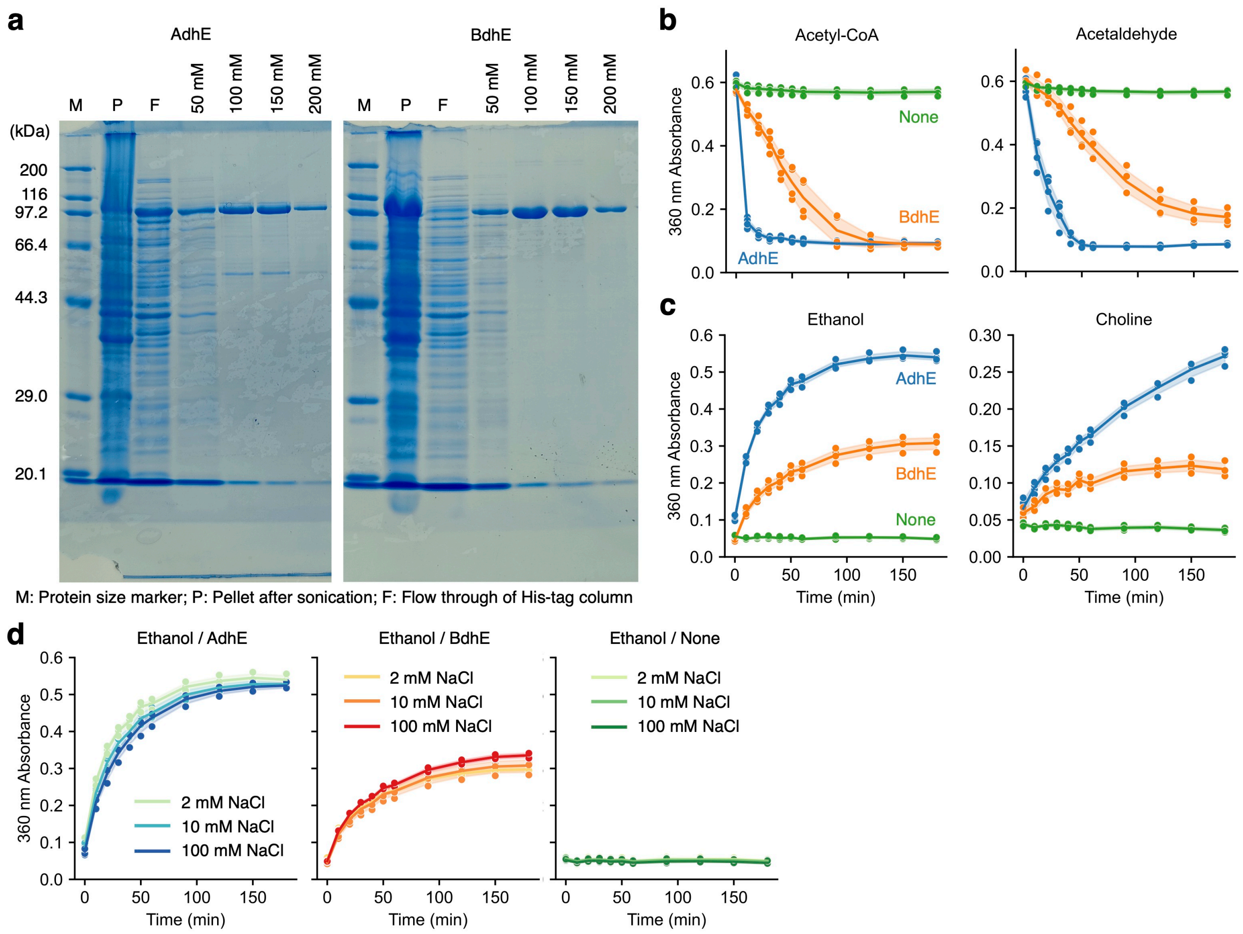

Supplementary Figure 4. Expression, purification, and enzymatic assays of AdhE and BdhE. a The results of sodium dodecyl sulfate-polyacrylamide gel electrophoresis (SDS-PAGE) for intermediates and final products of expression and His-tag purification of AdhE and BdhE. Lanes "M", "P", and "F" indicate molecular weight marker, the centrifugation pellet after sonication, and the flow through of His-tag purification. "50 mM" to "200 mM" indicate the eluate by adding various concentrations (50-200 mM) of imidazole. The molecular weights of AdhE and BdhE monomers with His-tag were 99.7 kDa and 97.9 kDa, respectively. **b, c** Enzymatic assays of acetyl-CoA/acetaldehyde oxidization (b) and ethanol/choline oxidization (c) by AdhE and BdhE purified by a His-tag affinity column and a size-exclusion column chromatography. The time-course absorbance of 360 nm was measured as concentrations of NADH for four replicates. "None" represents conditions in which only buffer without enzymes were added. **d** Ethanol oxidization assay under various NaCl concentration conditions.

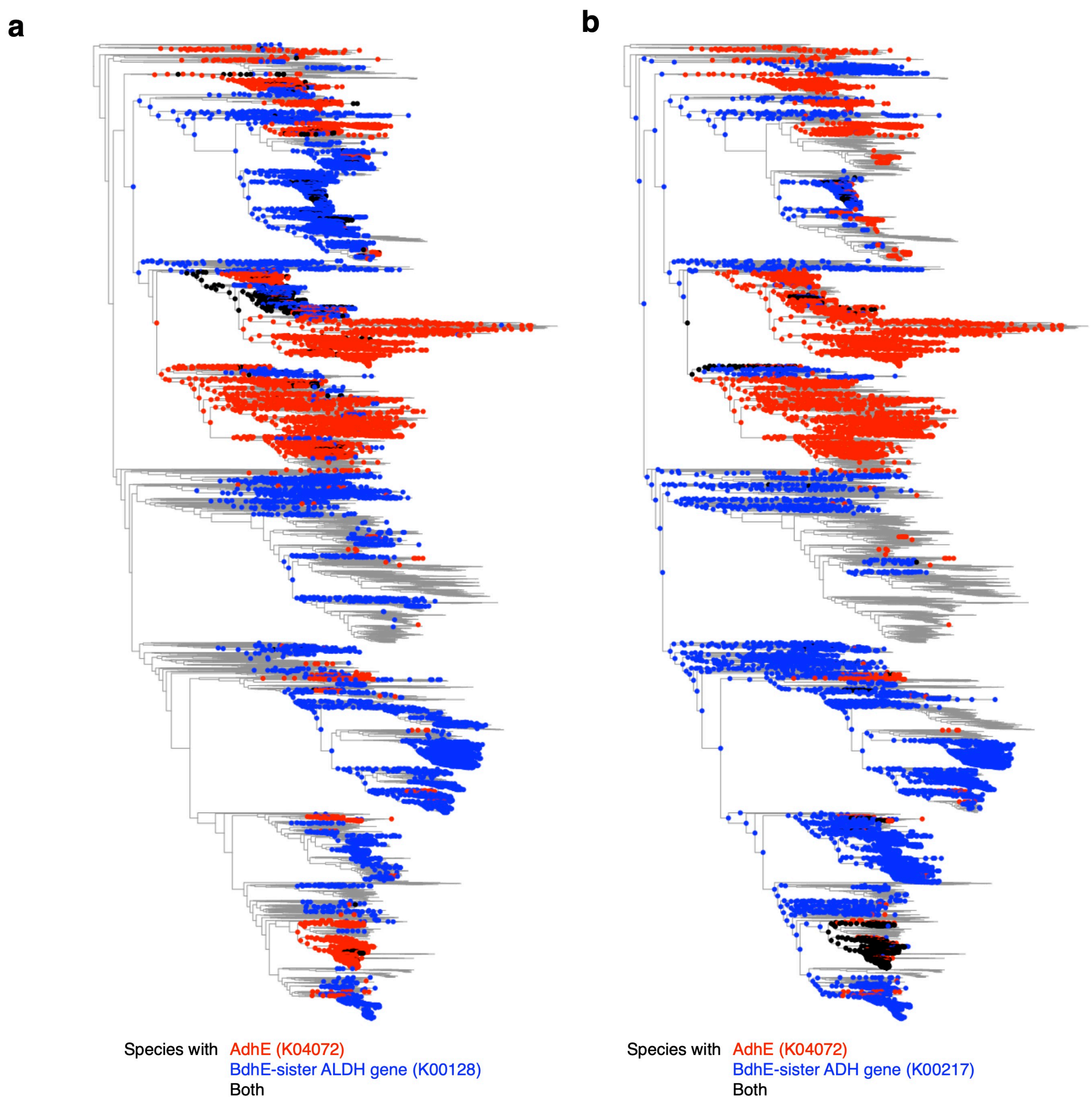

Supplementary Figure 5. Anti-correlated phylogenetic distributions of *adhE* and *bdhE*-sister genes. Phylogenetic distribution of *adhE* and *bdhE*-sister genes with ALDH (a) or ADH (b). The phylogenies are bacterial species trees of all the 25,877 species with high-quality genomes (>95% completeness and <5% contamination). The red, blue, and black nodes here indicate the extant/ancestral nodes estimated to possess AdhE, BdhE-sister proteins, and both, respectively. The ancestral states were estimated with PastML v.1.9.15.

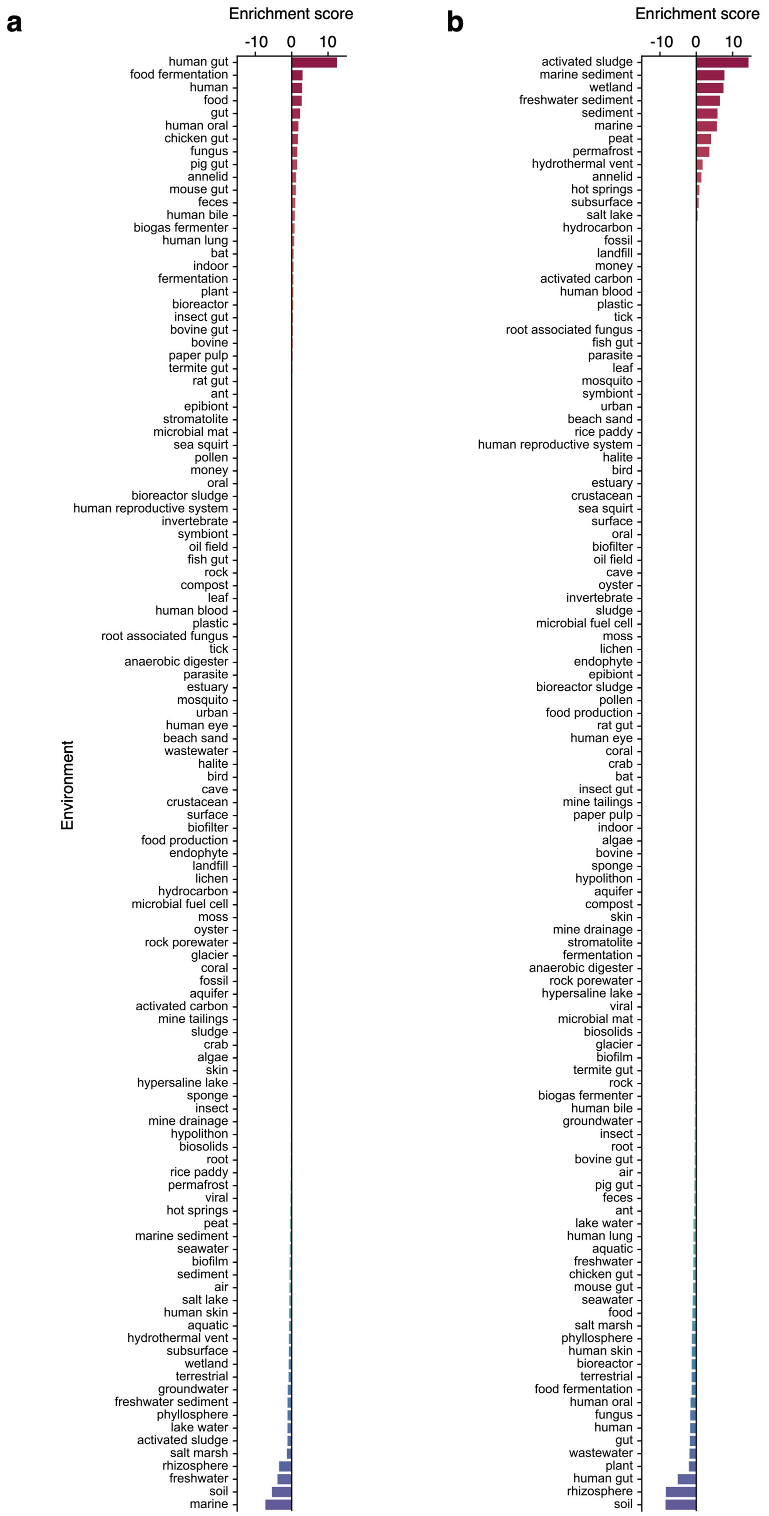

Supplementary Figure 6. Habitat preference of species possessing AdhE or BdhE. a, b The habitat enrichment of species with AdhE or BdhE. The enrichment score represents the difference between each environment's average habitat preference scores calculated for species with and without AdhE (a) or BdhE (b). The habitat preference scores were calculated using the ProkAtlas pipeline (see METHODS).

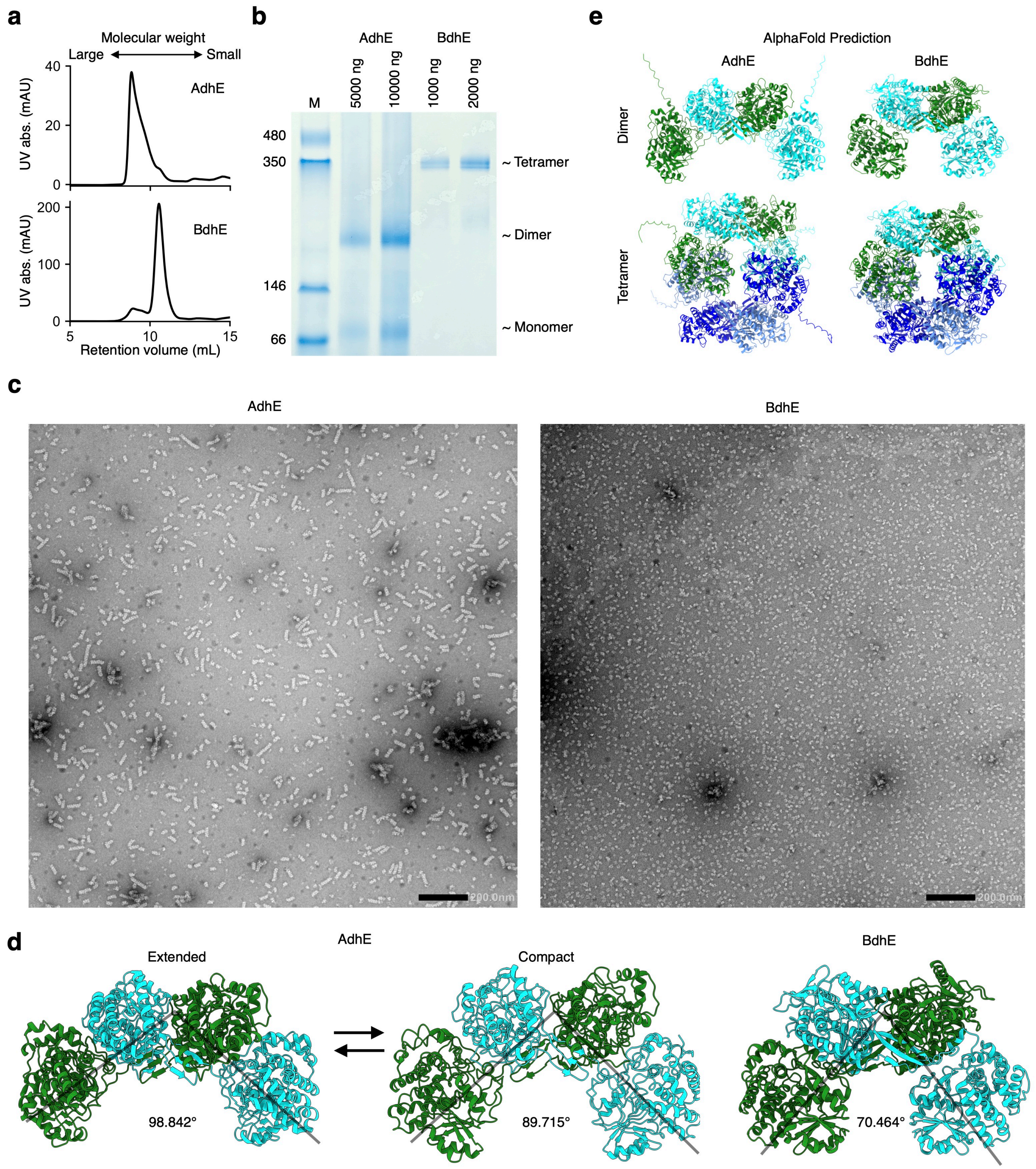

Supplementary Figure 7. Multimer stoichiometry and structural analysis for AdhE and BdhE. a Chromatograms of AdhE and BdhE by gel filtration chromatography after his-tag purification. **b** Blue native PAGE for AdhE and BdhE after his-tag purification. The applied weights of proteins in loaded samples are indicated above the gel. **c** The full negative stain electron microscopy analysis of AdhE and BdhE. Scale bar 200.0 nm. **d** The dimeric structure units of AdhE and BdhE. The bending angles of AdhE and BdhE represent angles formed by connecting C-α atoms at L638 and P208 of a chain, and L638 of the other chain in AdhE, F681 and K250 of a chain, and F681 of the other chain in BdhE, respectively. For AdhE, the angle was calculated for two distinct conformations (extended (PDB ID: 6TQH) and compact (PDB ID:

6TQM)). **e** Dimer and tetramer structures of AdhE and BdhE from *E. coli* and *H. eurihalina* predicted by AlphaFold v2.3.2. Different molecules are colored differently.

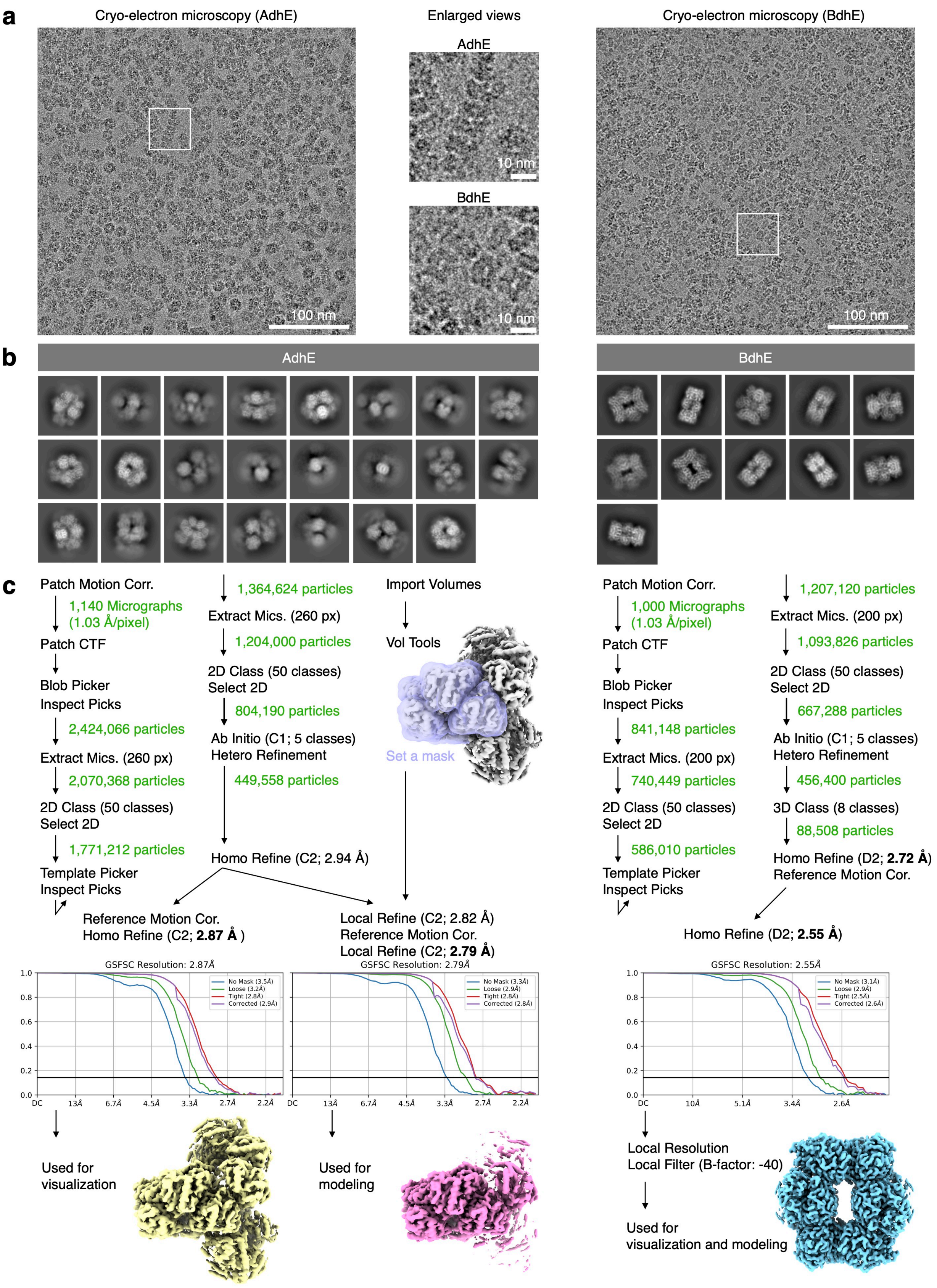
a
Cryo-electron microscopy (AdhE)
100 nm
Enlarged views
AdhE
10 nm
BdhE
10 nm
Cryo-electron microscopy (BdhE)
100 nm
b
AdhE
BdhE
c
Patch Motion Corr.
1,140 Micrographs
(1.03 Å/pixel)
Patch CTF
Blob Picker
Inspect Picks
2,424,066 particles
Extract Mics. (260 px)
2,070,368 particles
2D Class (50 classes)
Select 2D
1,771,212 particles
Template Picker
Inspect Picks
1,364,624 particles
Extract Mics. (260 px)
1,204,000 particles
2D Class (50 classes)
Select 2D
804,190 particles
Ab Initio (C1; 5 classes)
Hetero Refinement
449,558 particles
Homo Refine (C2; 2.94 Å)
Import Volumes
Vol Tools
Set a mask
Reference Motion Cor.
Homo Refine (C2; 2.87 Å)
Local Refine (C2; 2.82 Å)
Reference Motion Cor.
Local Refine (C2; 2.79 Å)
GSFSC Resolution: 2.87Å
No Mask (3.5Å)
Loose (3.2Å)
Tight (2.8Å)
Corrected (2.9Å)
GSFSC Resolution: 2.79Å
No Mask (3.3Å)
Loose (2.9Å)
Tight (2.8Å)
Corrected (2.8Å)
Used for
visualization
Used for
modeling
Patch Motion Corr.
1,000 Micrographs
(1.03 Å/pixel)
Patch CTF
Blob Picker
Inspect Picks
841,148 particles
Extract Mics. (200 px)
740,449 particles
2D Class (50 classes)
Select 2D
586,010 particles
Template Picker
Inspect Picks
1,207,120 particles
Extract Mics. (200 px)
1,093,826 particles
2D Class (50 classes)
Select 2D
667,288 particles
Ab Initio (C1; 5 classes)
Hetero Refinement
456,400 particles
3D Class (8 classes)
88,508 particles
Homo Refine (D2; 2.72 Å)
Reference Motion Cor.
Homo Refine (D2; 2.55 Å)
GSFSC Resolution: 2.55Å
No Mask (3.1Å)
Loose (2.9Å)
Tight (2.5Å)
Corrected (2.6Å)
Local Resolution
Local Filter (B-factor: -40)
Used for
visualization and modeling

Supplementary Figure 8. Workflows of cryo-electron microscopy analysis. a An example micrograph of cryo-electron microscopy for AdhE and BdhE. The enlarged view of each micrograph is shown in the middle, and the enlarged regions are shown with white boxes in the full original micrographs. In comparison with the negative stain image (**Supplementary Fig. 6c**), a larger proportion of AdhE particles appeared as top views rather than side views of filaments. This could be due to the random orientation of short AdhE filaments in the frozen sample for the cryo-electron microscopy experiments. **b** Reconstructed 2D images of AdhE and BdhE after 2D classification of particles. Each image corresponds to each particle class. **c** Pipelines to reconstruct electron density maps of AdhE and BdhE. CTF refinement was conducted in the homogeneous refinement process (Homo Refine).

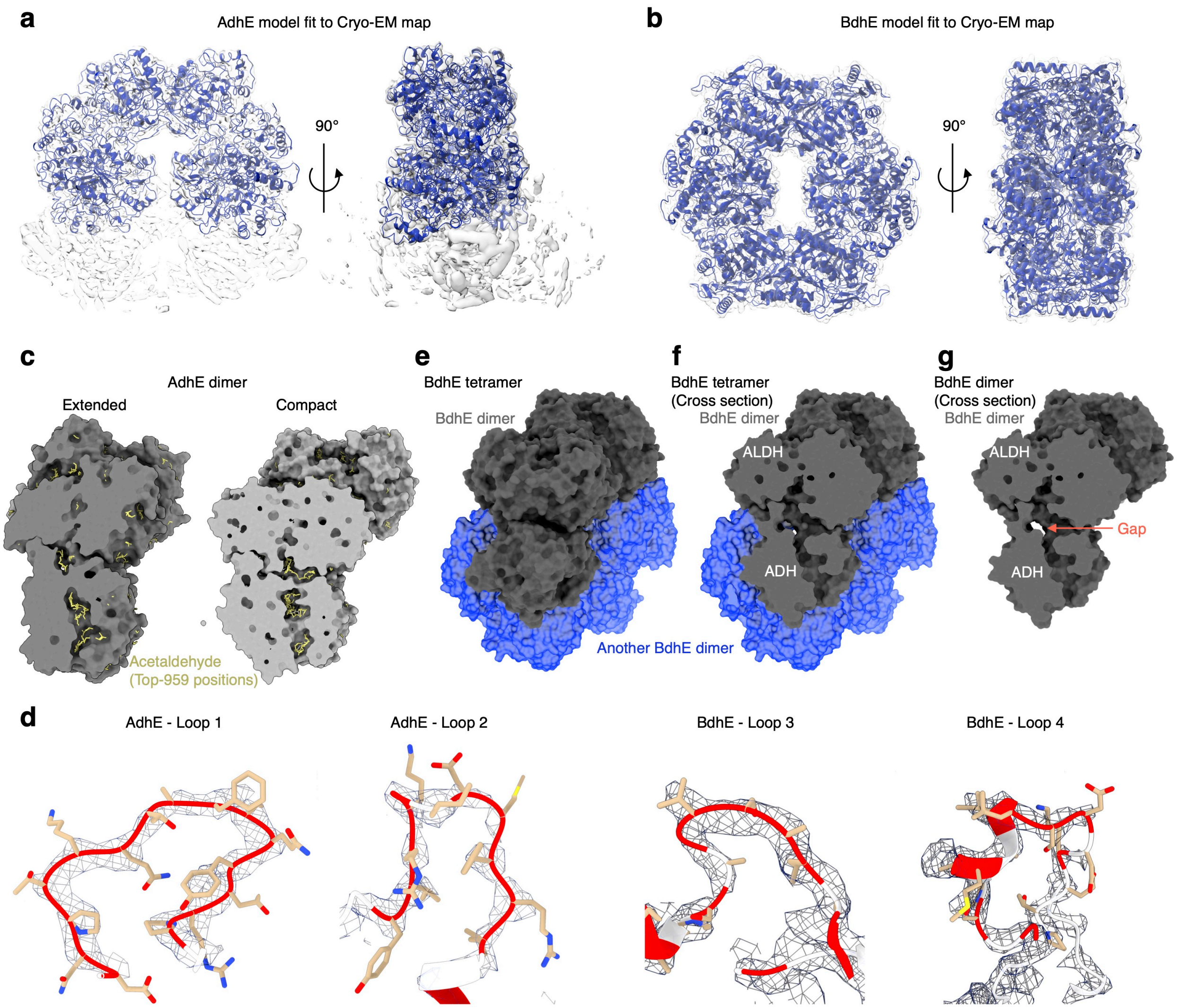

Supplementary Figure 9. Comparison of multimeric structures and inter-molecular interactions of AdhE and BdhE. a, b 3D molecular models of AdhE (a) and BdhE (b) fit to the corresponding Cryo-EM maps. **c** Docking simulation results of AdhE dimers and an acetaldehyde. Docking simulations were conducted for AdhE dimers of extended and compact conformation. The yellow molecular structures represent 959 possible docking positions of acetaldehyde within 4 kcal/mol from the lowest-energy position. **d** Structures of loops 1-4 in AdhE (extended conformation, PDB ID: 6TQH) or BdhE with electron density maps. Levels of electron density is 0.032 and 0.10 for AdhE and BdhE, respectively. **e-g** Gap filling of substrate tunnel wall in a BdhE dimer by another dimer in BdhE, respectively**.** The surface structure of BdhE tetramer (e), the cross-section of the tetramer (f), and a cross-section of single dimeric structure unit of BdhE (g). The structural gap of the substrate tunnel is indicated with an orange allow (g), but is partially filled by binding with another dimeric unit (f).

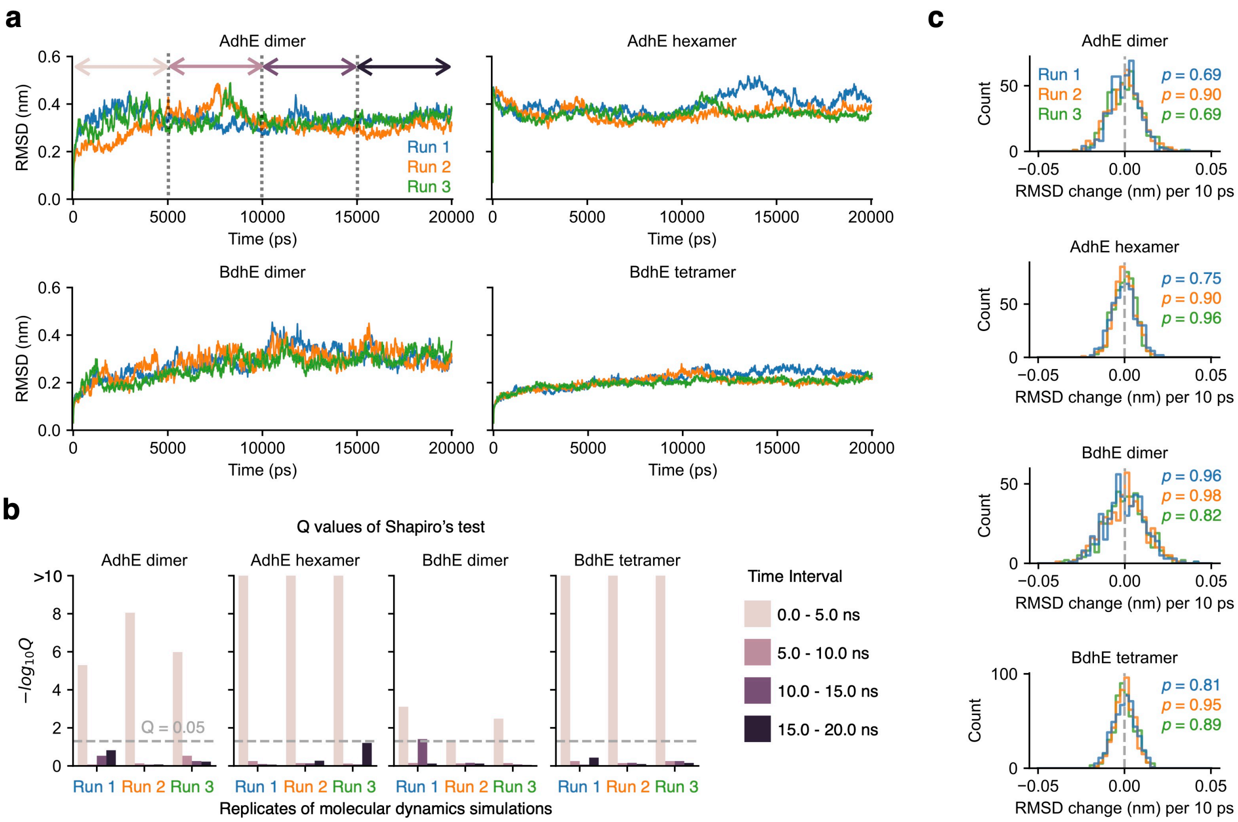

Supplementary Figure 10. Structural stability analysis of AdhE and BdhE by molecular dynamics simulations. a 20 ns time-course tracking of RMSD for AdhE and BdhE's structures while molecular dynamics simulations and their initial structures. Molecular dynamics simulation was conducted three times as replicate experiments (run 1-3). RMSD was calculated every 10 ps. The line plots with different colors correspond to different MD runs. The four bidirectional arrows in the top left plot indicates four different time intervals (0–5 ns, 5–10 ns, 10–15 ns, and 15–20 ns) analyzed in **b**. **b** The results of the Shapiro–Wilk test assessing the normality of RMSD change distributions (per 10 ps) within each of the four time intervals in each simulation. **c** RMSD change distributions (per 10 ps) for 15–20 ns time intervals in each simulation. The p-values of one-sample T-test for each distribution are shown as well.

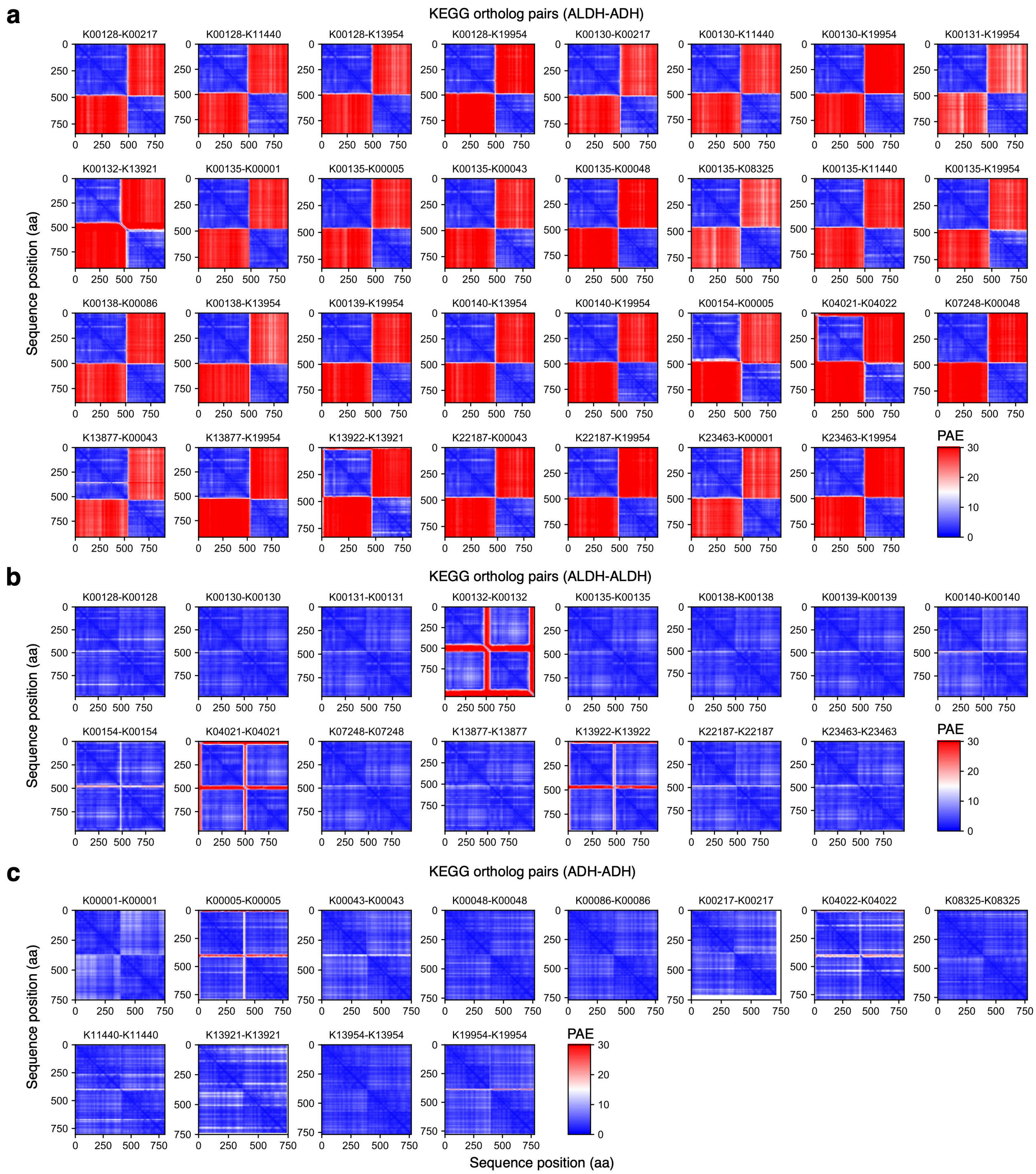

Supplementary Figure 11. ColabFold-predicted heteromeric and homomeric interactions of ALDH and ADH proteins. a ColabFold structure prediction of heteromeric interaction between ALDH and ADH genes adjacently coded in genomes. Structural predictions were done by ColabFold v1.5.2. The heatmap indicates the positioned aligned errors (PAEs). **b, c** ColabFold structure prediction of homodimeric interactions, i.e., ALDH-ALDH (b), and ADH-ADH (c) contacts.

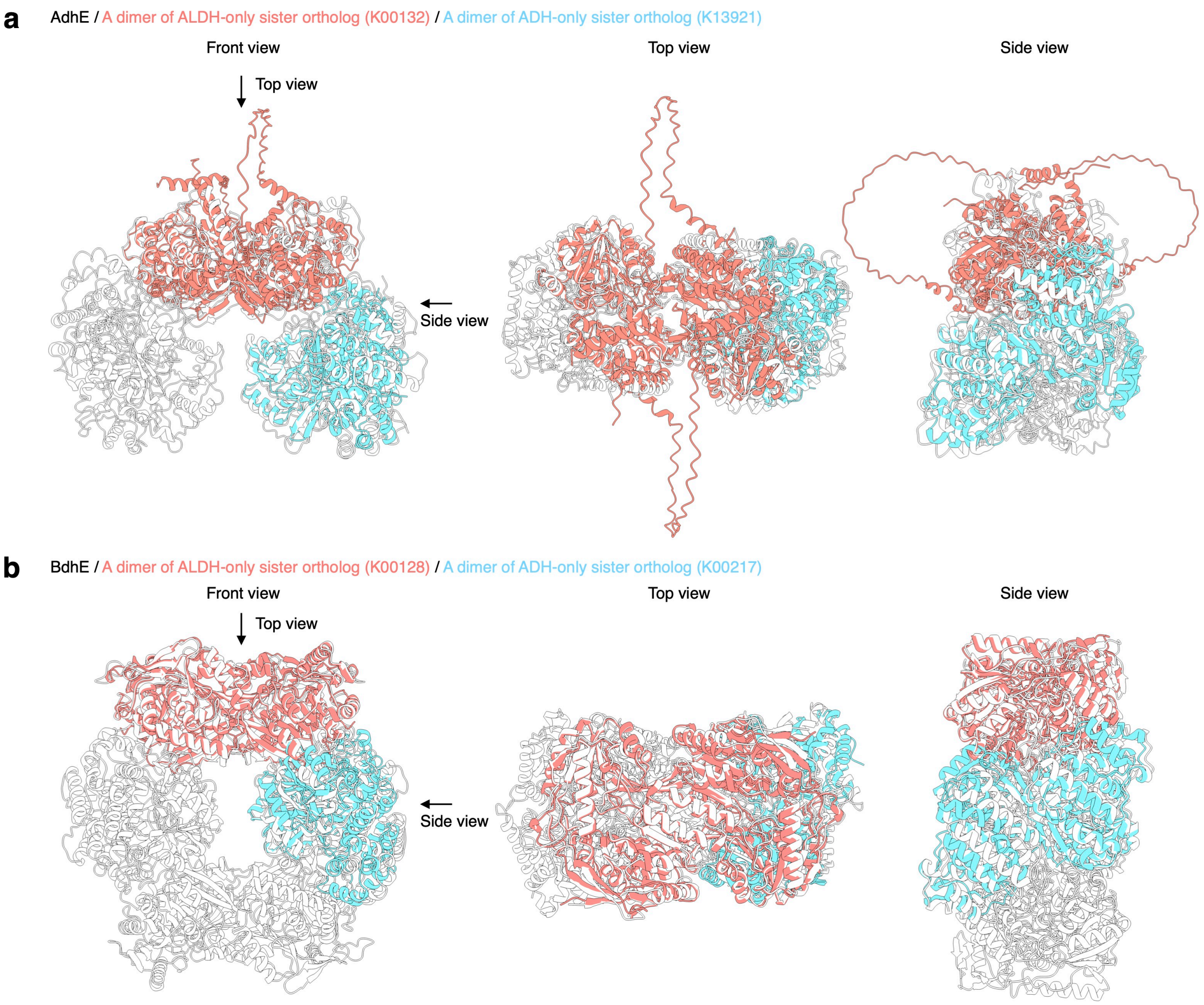

Supplementary Figure 12. Structural alignment of AdhE/BdhE and the predicted structures of the sister-clade proteins. a, b Three-dimensional structure of AdhE (a; PDB ID: 6TQM) or BdhE (b; solved in this study), aligned with a ColabFold-predicted dimeric structure of a randomly selected protein from the sister clade of AdhE or BdhE.

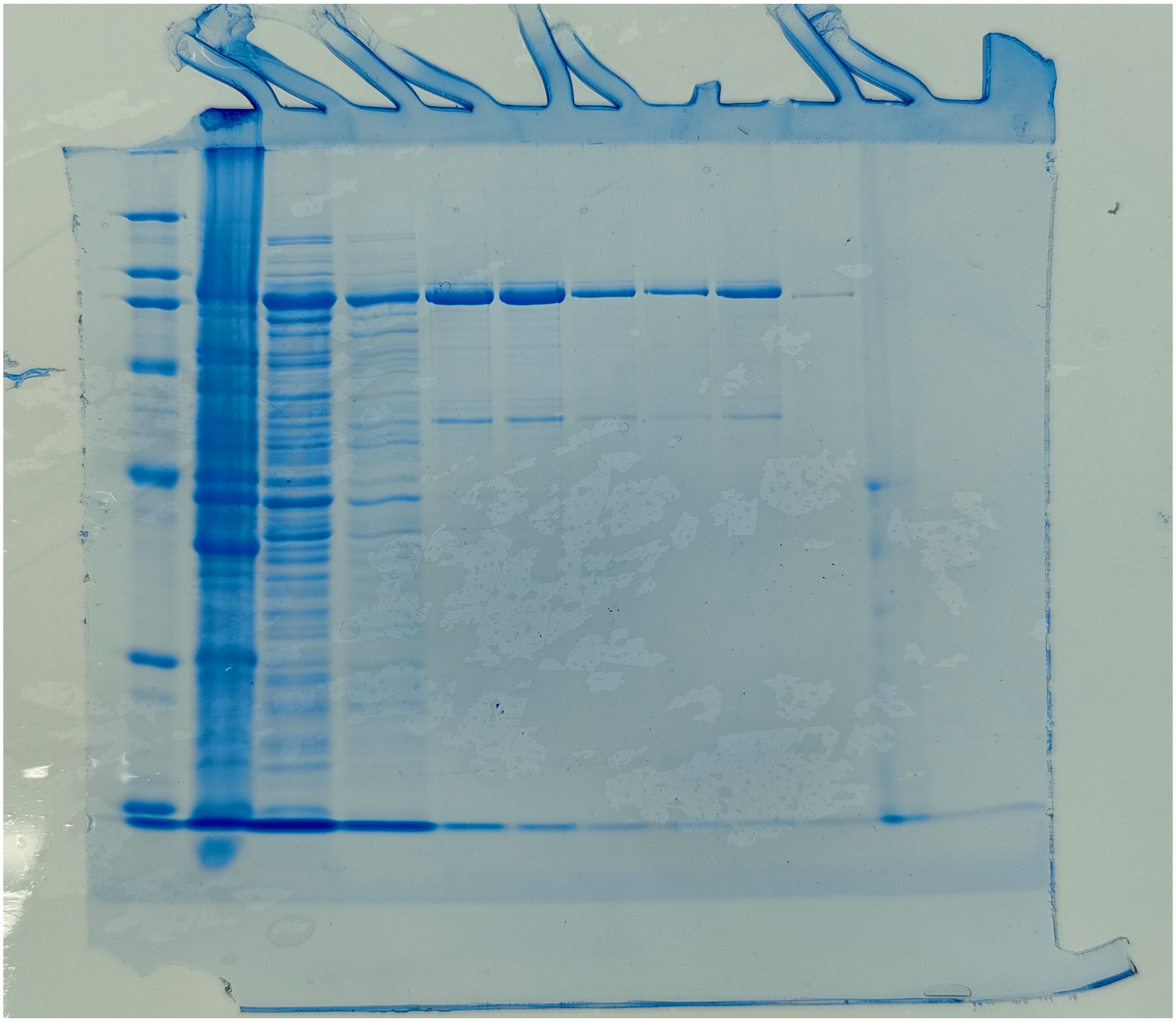

Supplementary Source Data 1. The original gel image for AdhE in Supplementary Fig. 4a. From left to right, the lanes correspond to the molecular weight marker, the centrifugation pellet after sonication, the flow-through from His-tag purification, the His-tag purification eluates of AdhE at 50, 100, 150, 200, 250 mM, and 1 M imidazole, and the His-tag purification eluate of BdhE at 1 M imidazole as a control.

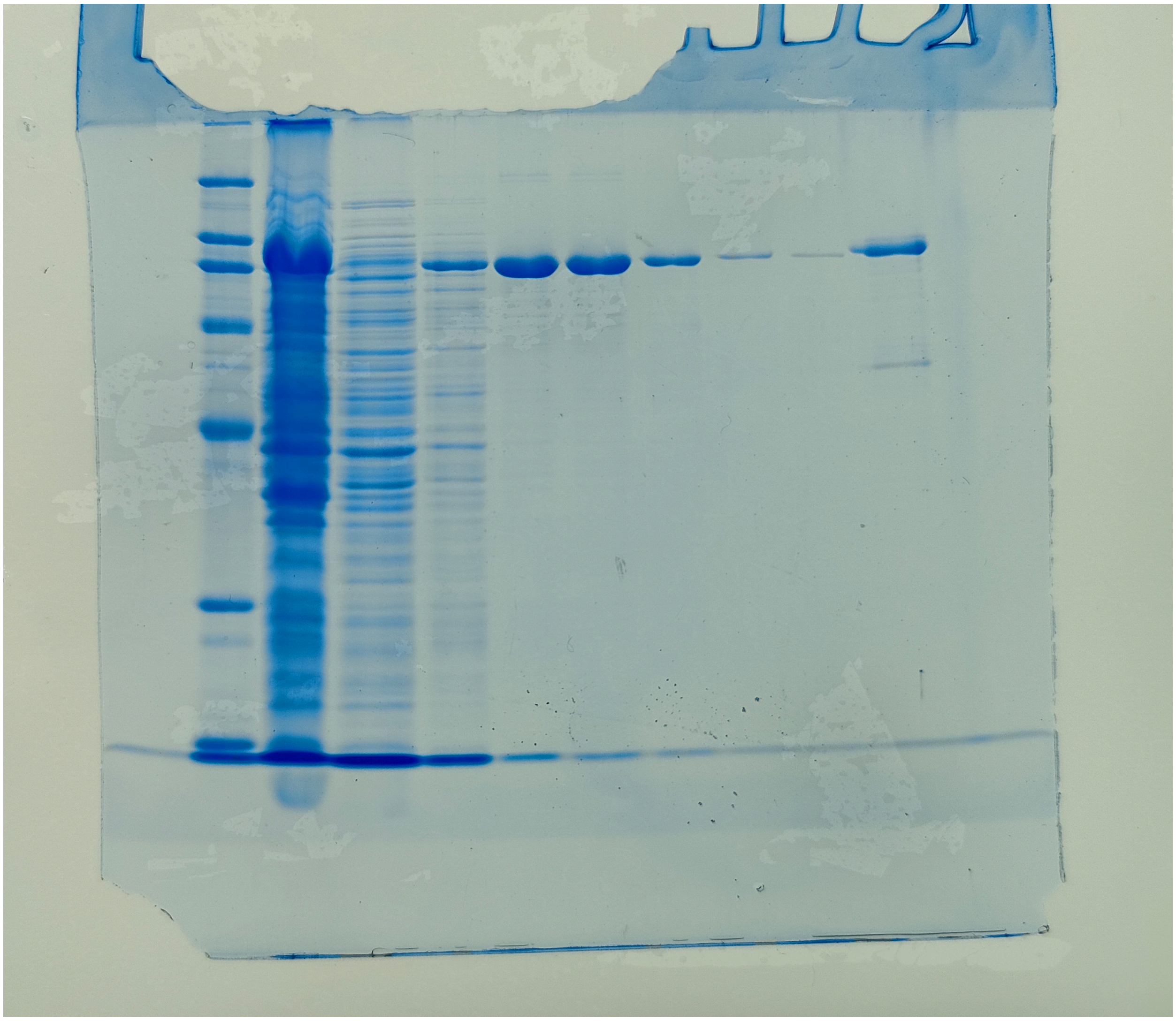

Supplementary Source Data 2. The original gel image for BdhE in Supplementary Fig. 4a. From left to right, the lanes correspond to the molecular weight marker, the centrifugation pellet after sonication, the flow-through from His-tag purification, the His-tag purification eluates of BdhE at 50, 100, 150, 200, 250 mM, and 1 M imidazole, and the His-tag purification eluate of AdhE at 1 M imidazole as a control.

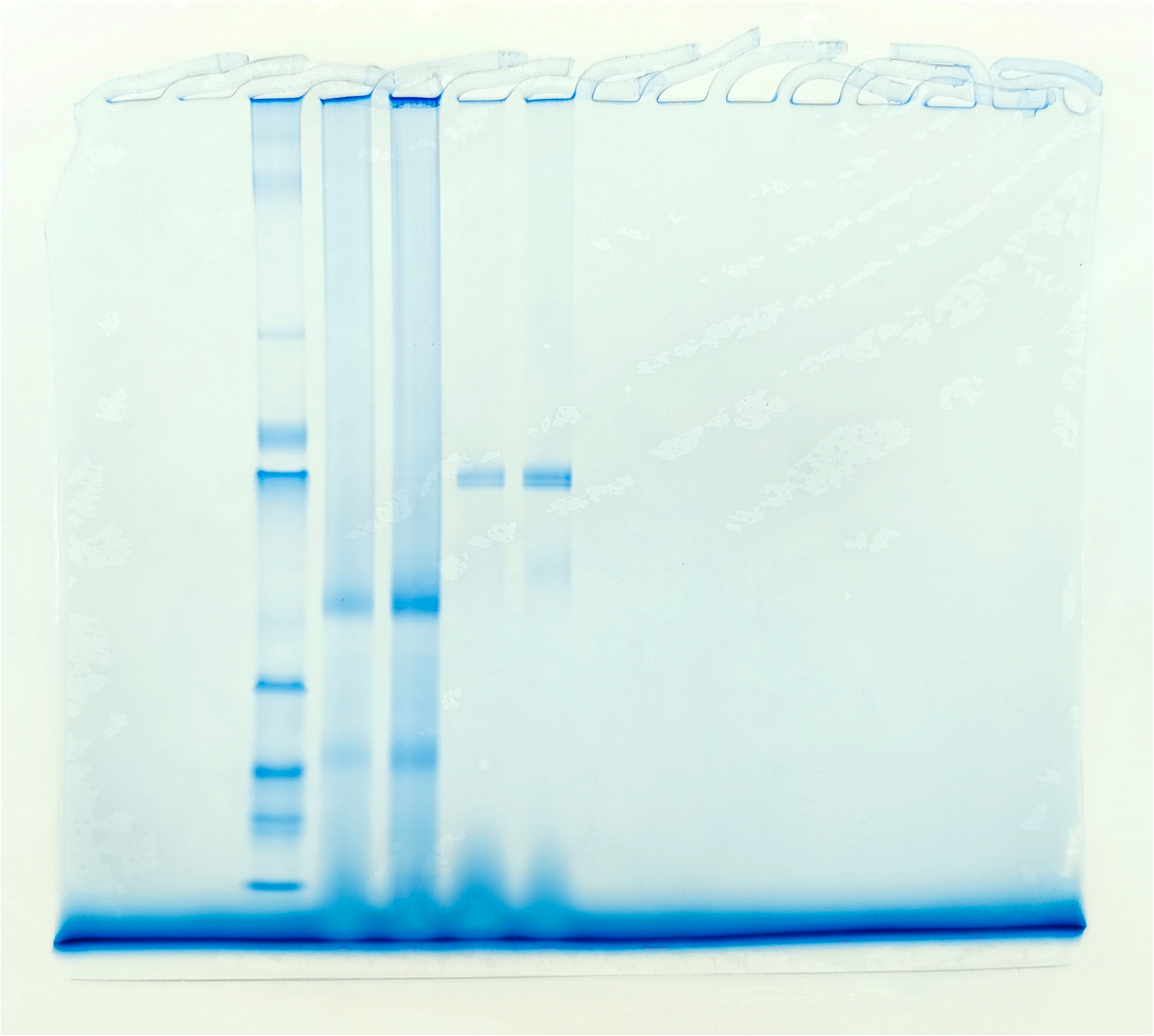

Supplementary Source Data 3. The original gel image for Supplementary Fig. 7b.

Supplementary Table 1. A table of Cryo-EM data collection, ref

Category	Value
Accession codes	PDB ID
Accession codes	EMDB ID
Data collection	Microscope
Data collection	Camera
Data collection	Voltage
Data collection	Magnification
Data collection	Electron exposure (e^-/Å^2)
Data collection	Pixel size (Å^2)
Data collection	Defocus range (μm)
Data collection	Processing
Data collection	Symmetry imposed
Data collection	Initial particle ranges (no.)
Data collection	Final particle ranges (no.)
Data collection	[illegible]
Refinement	[illegible]
Validation	RMSZ: Bond length
Validation	RMSZ: Bond angles
Validation	Ramachandran favored (%)
Validation	Ramachandran allowed (%)
Validation	Ramachandran outliers (%)
Validation	Rotamers outliers (%)
Validation	MolProbity: MolProbity score
Validation	MolProbity: Clashscore
Validation	Phenix: CC (mask)
Validation	Phenix: CC (box)
Validation	Phenix: CC (peaks)
Validation	Phenix: CC (volume)
Statistics	No. of chains
Statistics	Atoms (no.)
Statistics	Residues (no.)

inement, validation, and statistics

AdhE (PDB ID: 9LDK)	BdhE (PDB ID: 9LDL)
9LDK	9LDL
EMD-63003	EMD-63004
Talos Arctica G2	Talos Arctica G2
K2 direct ditector	K2 direct ditector
200 kV	200 kV
130,000	130,000
50	50
1.03	1.03
-1.75, -1.5, -1.25, -1.0	-1.75, -1.5, -1.25, -1.0
Single particle analysis	Single particle analysis
C2	D2
2,070,368	740,449
449,558	88,508
2.79	2.55
not applicable	-40
0.28	0.25
0.54	0.48
94	98
6	2
0	0
9	0
2.53	1.24
14.43	4.18
0.86	0.88
0.75	0.85
0.63	0.84
0.86	0.88
4	4
19,646	25,520
2,568	3,544

Supplementary Table 2. A table of molecular dynamics simulation box

Parameters	6AHC	6AHC dimer
box type	dodecahedron	dodecahedron
box vectors (nm)	20.949, 20.949, 20.949	16.261, 16.261, 16.261
box volume (nm^3)	6501.05	3040.59
Number of atoms	643385	299354
Number of water molecules	183525	90920
Number of Na+ molecules	82	22
Number of protein chains	8	2
salt concentration	neutralized only	neutralized only

‹ parameters.

BdhE tetramer	BdhE dimer
dodecahedron	dodecahedron
16.592, 16.592, 16.592	15.247, 15.247, 15.247
3230.02	2506.21
320158	248357
89026	73939
80	40
4	2
neutralized only	neutralized only

References

1. K. Katoh, K. Misawa, K.-I. Kuma, T. Miyata, MAFFT: a novel method for rapid multiple sequence alignment based on fast Fourier transform. *Nucleic Acids Res.* **30**, 3059–3066 (2002).

2. S. Capella-Gutiérrez, J. M. Silla-Martínez, T. Gabaldón, trimAl: a tool for automated alignment trimming in large-scale phylogenetic analyses. *Bioinformatics* **25**, 1972–1973 (2009).
