# Supplementary material for: Repeatability of protein structural evolution following convergent gene fusions
Source: Nat Commun. 2025 Sep 22;16:8278. doi: 10.1038/s41467-025-63898-x (PMC12454647; doi:10.1038/s41467-025-63898-x)
Supplement: Supplementary file 3 — Description of Additional supplementary files [file 41467_2025_63898_MOESM3_ESM.pdf]

Title: Supplementary Movie 1

Description: Movies of molecular dynamics for AdhE dimer, AdhE hexamer, BdhE dimer, and BdhE tetramer. Movies for Run 1 in the three replication experiments are provided.

Title: Supplementary Data 1

Description: Multiple sequence alignment of BdhE, AdhE, and sister-clade single domain families of them.

Title: Supplementary Data 2

Description: Multiple sequence alignments used for gene phylogeny reconstructions of ALDH-containing proteins and

Title: Supplementary Data 3

Description: Multiple sequence alignments used for gene phylogeny reconstructions of ADH-containing proteins
