# Supplementary material for: Repeatability of protein structural evolution following convergent gene fusions
Source: Nat Commun. 2025 Sep 22;16:8278. doi: 10.1038/s41467-025-63898-x (PMC12454647; doi:10.1038/s41467-025-63898-x)
Supplement: Supplementary file 8 — Reporting Summary [file 41467_2025_63898_MOESM8_ESM.pdf]

nature portfolio

Corresponding author(s): Naoki Konno

Last updated by author(s): May 30, 2025

Reporting Summary

Nature Portfolio wishes to improve the reproducibility of the work that we publish. This form provides structure for consistency and transparency in reporting. For further information on Nature Portfolio policies, see our Editorial Policies and the Editorial Policy Checklist.

Statistics

For all statistical analyses, confirm that the following items are present in the figure legend, table legend, main text, or Methods section.

n/a	Confirmed	
☐	☒	The exact sample size (*n*) for each experimental group/condition, given as a discrete number and unit of measurement
☐	☒	A statement on whether measurements were taken from distinct samples or whether the same sample was measured repeatedly
☐	☒	The statistical test(s) used AND whether they are one- or two-sided<br>*Only common tests should be described solely by name; describe more complex techniques in the Methods section.*
☐	☒	A description of all covariates tested
☐	☒	A description of any assumptions or corrections, such as tests of normality and adjustment for multiple comparisons
☐	☒	A full description of the statistical parameters including central tendency (e.g. means) or other basic estimates (e.g. regression coefficient) AND variation (e.g. standard deviation) or associated estimates of uncertainty (e.g. confidence intervals)
☐	☒	For null hypothesis testing, the test statistic (e.g. *F*, *t*, *r*) with confidence intervals, effect sizes, degrees of freedom and *P* value noted<br>*Give P values as exact values whenever suitable.*
☒	☐	For Bayesian analysis, information on the choice of priors and Markov chain Monte Carlo settings
☐	☒	For hierarchical and complex designs, identification of the appropriate level for tests and full reporting of outcomes
☒	☐	Estimates of effect sizes (e.g. Cohen's *d*, Pearson's *r*), indicating how they were calculated

Our web collection on statistics for biologists contains articles on many of the points above.

Software and code

Policy information about availability of computer code

Data collection	We retrieved all the protein sequences of every prokaryotic representative genome and reference phylogenies from GTDB r202 on April 28, 2021. We retrieved the structure models of the extended and compact form of AdhE from the Protein Data Bank (PDB ID: 6TQH, 6TQM, and 6AHC).
Data analysis	KofamScan v1.3.0, ete3 toolkit 3.1.2, MMseqs v13.45111, MAFFT v7.310, TrimAl v1.4rev15, IQTree v2.0.3, InterPro (retrieval date: November 29, 2022), ProkAtlas online accessed on June 30, 2021, CryoSPARC v4.4.1, Coot v0.9.8.91, UCSF ChimeraX v1.7rc202311290355, MolProbity (v.4.5.2), Phenix v1.20.1-4487, Openbabel v3.1.0, UCSF Chimera v1.17.3, AutoDock Vina v1.2.5, GROMACS (version 2023.2), CHARMM36m, PyMOL v2.5.4

For manuscripts utilizing custom algorithms or software that are central to the research but not yet described in published literature, software must be made available to editors and reviewers. We strongly encourage code deposition in a community repository (e.g. GitHub). See the Nature Portfolio guidelines for submitting code & software for further information.

Data

Policy information about availability of data

All manuscripts must include a data availability statement. This statement should provide the following information, where applicable:

- Accession codes, unique identifiers, or web links for publicly available datasets
- A description of any restrictions on data availability
- For clinical datasets or third party data, please ensure that the statement adheres to our policy

The atomic coordinates and cryo-EM density maps have been deposited in the PDB and the Electron Microscopy Data Bank (EMDB), respectively, under the following accession codes: 9LDK [https://doi.org/10.2210/pdb9LDK/pdb] (AdhE) and 9LDL [https://doi.org/10.2210/pdb9LDL/pdb] (BdhE) for PDB. EMD-63003 [https://www.ebi.ac.uk/pdbe/entry/emdb/ EMD-63003] (AdhE) and EMD-63004 [https://www.ebi.ac.uk/pdbe/entry/emdb/EMD-63004] (BdhE) for EMDB. The molecular dynamics result datasets are provided in Zenodo [https://doi.org/10.5281/zenodo.15322847]. The Source Data files for all data presented in graphs within each Figure are also provided as supplementary materials. The previously published PDB entries analysed in this study are available under the following accession codes: 6TQH [https://doi.org/10.2210/pdb6TQH/pdb] (E. coli AdhE structure in its extended conformation), 6TQM [https://doi.org/10.2210/pdb6TQM/pdb] (E. coli AdhE structure in its compact conformation), and 6AHC [https://doi.org/10.2210/pdb6AHC/pdb] (E. coli AdhE structure in its compact conformation).

Research involving human participants, their data, or biological material

Policy information about studies with human participants or human data. See also policy information about sex, gender (identity/presentation), and sexual orientation and race, ethnicity and racism.

Reporting on sex and gender	Not applicable
Reporting on race, ethnicity, or other socially relevant groupings	Not applicable
Population characteristics	Not applicable
Recruitment	Not applicable
Ethics oversight	Not applicable

Note that full information on the approval of the study protocol must also be provided in the manuscript.

Field-specific reporting

Please select the one below that is the best fit for your research. If you are not sure, read the appropriate sections before making your selection.

☐ Life sciences ☐ Behavioural & social sciences ☒ Ecological, evolutionary & environmental sciences

For a reference copy of the document with all sections, see nature.com/documents/nr-reporting-summary-flat.pdf

Ecological, evolutionary & environmental sciences study design

All studies must disclose on these points even when the disclosure is negative.

Study description	We conducted phylogenetic analysis of ALDH and ADH protein families, biochemically and structurally characterized a newly found a ALDH-ADH fusion enzyme.
Research sample	We sampled all the high-quality 25877 representative genomes from GTDB r202. We chose one representative protein for AdhE and BdhE to biochemically and structurally characterize.
Sampling strategy	We sampled all the high-quality 25877 representative genomes from GTDB r202. We chose representative AdhE and BdhE proteins possessed by evolutionarily close taxa
Data collection	Data collection of genomes was mainly conducted by downloading from a public database, GTDB r202. Wet lab experiment data was collected by the corresponding author, Naoki Konno. The molecular dynamics data was collected by Mr. Satoshi Nishino
Timing and spatial scale	Not applicable
Data exclusions	Not applicable
Reproducibility	We prepared three or more replicates for each enzymatic assay and molecular dynamics experiments.
Randomization	Not applicable
Blinding	Not applicable. All the available data with were collected from the GTDB r207 and analyzed with phylogeny-based group allocation.

Did the study involve field work? ☐ Yes ☒ No

Reporting for specific materials, systems and methods

We require information from authors about some types of materials, experimental systems and methods used in many studies. Here, indicate whether each material, system or method listed is relevant to your study. If you are not sure if a list item applies to your research, read the appropriate section before selecting a response.

Materials & experimental systems

n/a	Involved in the study
☒	☐ Antibodies
☒	☐ Eukaryotic cell lines
☒	☐ Palaeontology and archaeology
☒	☐ Animals and other organisms
☒	☐ Clinical data
☒	☐ Dual use research of concern
☒	☐ Plants

Methods

n/a	Involved in the study
☒	☐ ChIP-seq
☒	☐ Flow cytometry
☒	☐ MRI-based neuroimaging

Plants

Seed stocks	Not applicable
Novel plant genotypes	Not applicable
Authentication	Not applicable
